# Sycosis

**Published:** 1893-07

**Authors:** A. McNeil

**Affiliations:** San Francisco, Cal.


### SYCOSIS.

A. McNeil, M. D., San Francisco, Cal.

For several years our conviction has been becoming firmer that the greatest number of the urinary complaints and those of the uro-poetic organs grow out of sycosis, or what we hold to be identical pock (variola) and can only be permanently cured by such remedies as correspond to this constitutional primeval taint. The number of these remedies is far greater, however, than is generally believed, and many of the so-called anti-psorics but particularly Ant-crud., Ant-tart., Arg-met., Arsen., Aur., Bar-carb., Bell., Calc-carb., Carb-anim., Carb-veg., Caust., Euphras., Jod., Lach., Lycop., Mercur., Mur-ac., Nit-ac., Nux-vom., Phos-ac., Puls., Rhus, Sabin., Secal-cor., Selen, Sep., Silic., Staph., Sulph., Zinc, have demonstrated their curative virtues in these different morbid manifestations. It is to be expected, therefore, that in future Homœopathy will make considerable progress in the treatment of these difficult or incurable diseases.

